# Urinary Biomarkers of Polycyclic Aromatic Hydrocarbons Are Associated with Cardiometabolic Health Risk

**DOI:** 10.1371/journal.pone.0137536

**Published:** 2015-09-04

**Authors:** Mahsa Ranjbar, Michael A. Rotondi, Chris I. Ardern, Jennifer L. Kuk

**Affiliations:** School of Kinesiology and Health Science, York University, Toronto, Ontario, Canada; NSYSU, TAIWAN

## Abstract

**Background:**

Polycyclic aromatic hydrocarbons (PAH) are both man-made and naturally occurring environmental pollutants that may be related to cardiometabolic health risk.

**Objective:**

To determine whether PAH is associated with obesity in the adult population and to examine whether urinary concentrations of PAH metabolites are associated with differences in how obesity relates to 3 or more risk factors for the metabolic syndrome (3RFMetS), type 2 diabetes (T2D), hypertension, and dyslipidemia.

**Methods:**

A total of 4765 adult participants from the 2001–2008 National Health and Nutrition Examination Survey were examined. The association between 8 urinary hydroxylated PAH metabolites, obesity, and health were examined using weighted logistic regressions adjusting for age, sex, ethnicity, PIR, smoking status, and urinary creatinine.

**Results:**

There was a positive dose-dependent association between obesity and 2-phenanthrene quintiles (P trend <0.0001). Contrarily, higher quintiles of 1-naphthalene were associated with lower risk of obesity (P trend = 0.0004). For a given BMI, those in the highest quintile of 2-naphthalene, 2-fluorene, 3-fluorene and 2-phenanthrene had a 66–80% greater likelihood of 3RFMetS (P≤0.05) compared to low levels. Higher quintiles of 1-naphthalene, 2-naphthalene, 2-phenanthrene and 1-pyrene were associated with a 78–124% greater likelihood of T2D (P≤0.05) compared to low levels while high 1-naphthalene, 2-naphthalene, 2-fluorene, 3-fluorene and 2-phenanthrene were associated with a 38–68% greater likelihood of dyslipidemia (P≤0.05) compared to lower levels. Finally, 2-naphthalene and 2-phenanthrene were positively associated with hypertension (P trend = 0.008 and P trend = 0.02 respectively).

**Conclusions:**

PAH is related to obesity and the expression of a number of obesity-related cardiometabolic health risk factors. Future research is needed to bring to light the mechanistic pathways related to these findings.

## Introduction

Polycyclic aromatic hydrocarbons (PAH) are man-made environmental pollutants resulting from the incomplete combustion of oil, fossil fuel, gas, coal and waste, and are also produced naturally from volcanoes and forest fires [1]. PAHs are used in many different commercial processes. For example, fluorene and phenanthrene are used in the production of dyestuffs while naphthalene is used in the production of sulfonates, insecticides and pharmaceuticals [2]. If not exposed to PAHs through ones occupation, the most common source of exposure is through the inhalation of particulates in the air. Alternatively, one can be exposed to PAH through the skin, ingestion of charred/smoked meat, or through the ingestion of contaminated produce and grains cultivated from polluted soil [1]. The majority of PAH are excreted from the body shortly after exposure, while small amounts are believed to be retained in fat and liver tissues [1,3]. In addition, it is suggested that PAH may accumulate in the body over time [3].

PAHs are known to be associated with cancer [4–10], and were recently linked with inflammation [11,12], fatal ischemic heart disease [13], peripheral arterial disease [14] and cardiovascular disease [15]. A recent study of PAH in children demonstrated a positive correlation between high concentrations of PAH and level of obesity [16]. However, it is unknown whether this environmental pollutant is related with obesity in adults and whether the associations between PAH and cardiometabolic conditions remain independent of BMI.

Therefore the objectives of this study are to determine whether PAH is related with obesity and to investigate whether the association between PAH and metabolic syndrome (MetS), hypertension, diabetes, and dyslipidemia is present, independent of BMI.

## Research Methods

### Participants

Adult participants at least 20 years of age from the National Health and Nutrition Examination Survey (NHANES; 2001–2008 survey years) with available hydroxylated PAH data were included in this study. NHANES is a nationally representative survey of the non-institutionalized United States (U.S.) population with microdata available online to the public [17]. Participants across the U.S. were selected using a complex processes involving Census information [17]. Certain minority groups such as Hispanics, the elderly, and low-income populations were oversampled to provide stable estimates for these study populations. The aim of the survey was to recruit a sample that could be weighted to be representative of the U.S. population. NHANES was granted ethics approval by the National Centre for Health Statistic Ethics Review Board. All individuals provided written informed consent prior to study participation. This study is a secondary analysis of anonymized data that did not require ethics approval from the institutional review board.

Participants were excluded from the study if they were pregnant (n = 274), had a BMI ≥ 70 kg/m^2^ (n = 2) or had missing data for BMI (n = 98), poverty income ratio (PIR, n = 354), or smoking status (n = 6). Individuals who could not be categorized into having or not having MetS (n = 189) or dyslipidemia (n = 111) due to missing data were also excluded resulting in 4765 remaining participants.

### Polycyclic Aromatic Hydrocarbons

After a home interview was conducted, participants were given a second examination at a mobile examination center (MEC). Spot-urine samples were randomly collected from a subsample consisting of a third of the participants. Trained professionals at the MEC collected the urine samples from participants and stored them at-20°C or lower for transportation. Capillary gas chromatography as well as high-resolution mass spectrometry was used to quantify the metabolites of interest. The laboratory procedure for the measurement of PAH metabolites is described in great detail by NHANES [18].

A total of eight hydroxylated urinary PAH metabolites were observed including 1-hydroxynaphthalene (1-naphthalene), 2-hydroxynaphthalene (2-naphthalene), 2-hydroxyfluorene (2-fluorene), 3-hydroxyfluorene (3-fluorene), 1-hydroxyphenanthrene (1-phenanthrene), 2-hydroxyphenanthrene (2-phenanthrene), 3-hydroxyphenanthrene (3-phenanthrene), and 1-hydroxypyrene (1-pyrene). For this study, each PAH metabolite was categorized into weighted quintiles.

### Metabolic Syndrome, Dyslipidemia, Type 2 Diabetes and Hypertension

MetS was defined using the National Heart, Lung, and Blood Institute’s updated NCEP ATP III definition [19]. Individuals with three or more of the following four metabolic risk factors were classified as having 3RFMetS (3+ risk factor of MetS). These risk factors included: 1) cholesterol abnormalities (HDL ≤1.03 mmol/L for men and ≤1.29 mmol/L for women, were on cholesterol medication, or had a doctor diagnose them with abnormal cholesterol levels), 2) high triglyceride levels (≥1.69 mmol/L), 3) high blood pressure (systolic blood pressure (SBP) of ≥130 mm Hg, diastolic blood pressure (DBP) ≥ 85 mm Hg, taking hypertension medication, or had doctor diagnosed hypertension), or 4) blood glucose abnormalities (fasting glucose levels of ≥ 5.6 mmol/L, taking diabetes medication, taking insulin, or had doctor diagnosed diabetes). Waist circumference was excluded as one of the possible risk factors to avoid having obesity included as both a dependent and independent variable and allow for the examination of BMI as an independent variable.

Dyslipidemia was defined as: serum triglyceride levels ≥ 2.06 mmol/L, total cholesterol ≥ 6 mmol/L, HDL < 1.04 mmol/L for men and < 1.29 for mmol/L for women, were on cholesterol medication, or had doctor diagnosed hypercholesterolemia. Diabetes was defined as: fasting plasma glucose ≥ 7 mmol/L [20], plasma glycoheomoglobin (HbA1c) levels ≥ 6.5% [21], doctor diagnosed T2D, taking diabetes medication, or were taking insulin. Hypertension was defined as: SBP ≥ 140 mmHg, DBP ≥ 90 mmHg, doctor diagnosed hypertension, or taking hypertension medication [22].

### Statistical Analysis

Differences in participant characteristics by obesity status were examined using chi-square tests for categorical variables and t-tests for continuous variables. The prevalence (N,%) was presented for categorical variables, while the mean ± standard error (SE) was presented for continuous variables. Logistic regressions were performed to study the association between obesity and PAH quintiles adjusting for age, sex, ethnicity, PIR, smoking status and urinary creatinine. It should be noted that due to the high correlation between PAH and smoking [23,24], in addition to other covariates, all multivariable analysis was adjusted for the smoking status variable to account for the possible disadvantageous metabolic outcomes resulting from smoking status.

Adjusted logistic regression was performed to examine the association between 3RFMetS, hypertension, T2D, dyslipidemia and PAH respectively. Each regression model included an interaction term between PAH quintiles and BMI, if no significant interaction was observed, the term was removed and the main effects were examined. If a significant interaction was observed, BMI was categorized into normal weight, overweight and obese to facilitate graphing of the relationship between PAH and metabolic conditions by weight status. All statistical analyses were weighted to be representative of the general U.S. population using SAS v. 9.4 survey procedures. Significance was set at P ≤ 0.05.

## Results

**Table 1**presents the participant characteristics by obesity status. A total of 1680 (35.3%) individuals were defined as being obese (BMI ≥ 30 kg/m^2^). In general, obese individuals were significantly older (P < 0.0001) and had higher urinary creatinine levels (P < 0.0001).

**Table 1:** Participant characteristic and obesity status.

	Not Obese (n = 3085)	Obese (n = 1680)	*P*
Age (years)	45.1 ± 0.36	47.2 ± 0.45	<0.0001
BMI (kg/m^2^)	24.7 ± 0.06	35.5 ± 0.18	<0.0001
Urinary Creatinine (mg/dL)	122.21 ± 1.87	139.96 ± 2.58	<0.0001
Sex, *n* (% male)	1626 (52.7)	790 (47.02)	0.7112
Ethnicity, *n* (%)			<0.0001
White	1634 (54.0)	791 (47.1)	
Black	547 (17.7)	451 (26.8)	
Other	904 (29.3)	438 (26.1)	
Smoker, *n* (%)			0.0003
Never	1531 (49.6)	899 (53.5)	
Current	799 (25.9)	320 (19.0)	
Past	755 (24.5)	461 (27.4)	
PIR, *n* (% below poverty)	535 (17.3)	295 (17.6)	0.6112
3+ Risk Factor Metabolic Syndrome, *n* (%)	411 (13.3)	521 (31.0)	<0.0001
Hypertension, *n* (%)	1009 (32.7)	863 (31.0)	<0.0001
Type 2 Diabetes, *n* (%)	244 (7.9)	364 (21.7)	<0.0001
Dyslipidemia *n*, (%)	1680 (54.5)	1222 (72.7)	<0.0001
*PAH metabolites* (ng/L)*			
1-naphthalene	2823 ± 12	2675 ± 174	0.0018
2-naphthalene	3270 ± 139	3805 ± 174	0.2612
2-fluorene	322 ± 13	354 ± 15	0.8995
3-fluorene	136 ± 6	132 ± 7	0.1126
1-phenanthrene	144 ± 4	167 ± 5	0.0801
2-phenanthrene	59 ± 2	75 ± 3	<0.0001
3-phenanthrene	104 ± 3	106 ± 4	0.8705
1-pyrene	78 ± 3	85 ± 4	0.9650

Abbreviations: BMI, body mass index; PAH: polycyclic aromatic hydrocarbons; PIR, poverty index ratio

*PAH is presented as geometric mean ± SE; Age, BMI and urinary creatinine are presented as mean ± SE; Obesity is defined as BMI ≥ 30kg/m^2^

As can be seen in **Table 2**, a dose-dependent association was observed wherein higher quintiles of 2-phenanthrene was associated with a greater odds ratio for prevalent obesity (P_trend_ < 0.0001) after adjustment for age, sex, ethnicity, PIR, smoking status, and urinary creatinine. Conversely, a negative association was observed between 1-naphthalene quintiles and obesity (P_trend_ = 0.0004).

**Table 2:** Weighted and adjusted odds ratios for quintiles of PAH metabolites and obesity.

	Quintile 1	Quintile 2	Quintile 3	Quintile 4	Quintile 5	*P*_trend_
*Metabolite*:		OR (*95% CI*)	OR (*95% CI*)	OR (*95% CI*)	OR (*95% CI*)	
1-naphthalene	1 (referent)	1.00 (0.78, 1.29)	**0.70 (0.55, 0.90)**	**0.69 (0.52, 0.92)**	**0.66 (0.49, 0.88)**	0.0004
2-naphthalene	1 (referent)	0.99 (0.76, 1.30)	1.25 (0.99, 1.58)	**1.40 (1.10, 1.79)**	1.12 (0.86, 1.45)	0.02
2-fluorene	1 (referent)	1.13 (0.88, 1.46)	1.26 (0.99, 1.59)	1.08 (0.82, 1.42)	1.21 (0.92, 1.59)	0.34
3-fluorene	1 (referent)	0.94 (0.74, 1.20)	1.01 (0.80, 1.30)	0.82 (0.64, 1.05)	0.78 (0.60, 1.01)	0.04
1-phenanthrene	1 (referent)	1.24 (0.96, 1.59)	1.17 (0.91, 1.49)	1.14 (0.87, 1.50)	1.20 (0.91, 1.58)	0.41
2-phenanthrene	1 (referent)	**1.34 (1.02, 1.75)**	**1.66 (1.22, 2.26)**	**1.67 (1.21, 2.30)**	**1.79 (1.34, 2.40)**	<0.0001
3-phenanthrene	1 (referent)	1.04 (0.81, 1.32)	0.93 (0.69, 1.24)	0.81 (0.59, 1.10)	0.75 (0.55, 1.02)	0.02
1-pyrene	1 (referent)	1.11 (0.88,1.41)	1.186 (0.94, 1.50)	1.15 (0.90, 1.47)	1.10 (0.82, 1.47)	0.54

Obesity defined as BMI ≥ 30kg^2^; Weighted and adjusted for age, sex, poverty index ratio, ethnicity, smoking status, and urinary creatinine

**Table 3**demonstrates the association between the various PAH metabolites, 3RFMetS, and hypertension after adjusting for confounders. There were no significant interaction effects between PAH and BMI (P > 0.05) for 3RFMetS. There was a positive association between 3FMetS and quintiles of 2-naphthalene, 1-phenanthrene and 2-phenanthrene, wherein higher levels of the metabolites were associated with significantly greater risk of prevalent 3FMetS (P_trend_ = 0.008, P_trend_ = 0.05, and P_trend_ = 0.001 respectively). In specific, for a given BMI, those in the highest quintiles of 2-naphthalene, 2- and 3-fluorene and 2-phenanthrene had a 66–80% greater odds ratio for prevalent 3RFMetS when compared to the lowest quintile.

**Table 3:** Weighted and adjusted odds ratios for quintiles of PAH and 3+ factor metabolic syndrome and hypertension.

	3 Factors of Metabolic Syndrome					
	Quintile 1	Quintile 2	Quintile 3	Quintile 4	Quintile 5	*P*_trend_
*Metabolite*:		OR (*95% CI*)	OR (*95% CI*)	OR (*95% CI*)	OR (*95% CI*)	
1-naphthalene	1 (referent)	1.10 (0.82, 1.48)	0.80 (0.53, 1.23)	0.98 (0.64, 1.51)	1.23 (0.82, 1.92)	0.54
2-naphthalene	1 (referent)	1.16 (0.82, 1.64)	1.24 (0.87, 1.75)	1.41 (0.97, 2.06)	**1.76 (1.19, 2.60)**	0.008
2-fluorene	1 (referent)	1.22 (0.91, 1.62)	1.27 (0.87, 1.87)	1.09 (0.75, 1.60)	**1.66 (1.05, 2.62)**	0.11
3-fluorene	1 (referent)	**1.35 (1.02, 1.80)**	1.31 (0.93, 1.83)	0.94 (0.67, 1.30)	**1.80 (1.25, 2.59)**	0.12
1-phenanthrene	1 (referent)	1.22 (0.91, 1.62)	**1.49 (1.05, 2.11)**	**1.66 (1.15, 2.41)**	1.39 (0.94, 2.07)	0.05
2-phenanthrene	1 (referent)	1.30 (0.94, 1.79)	**1.59 (1.12, 2.28)**	**1.80 (1.30, 2.50)**	**1.74 (1.20, 2.52)**	0.001
3-phenanthrene	1 (referent)	**1.35 (1.06, 1.72)**	1.10 (0.82, 1.48)	1.29 (0.92, 1.80)	1.16 (0.85, 1.57)	0.58
1-pyrene	1 (referent)	0.70 (0.94, 1.52)	1.15 (0.79, 1.67)	1.13 (0.84, 1.53)	1.09 (0.74, 1.60)	0.15
	**Hypertension**					
1-naphthalene	1 (referent)	1.02 (0.75, 1.34)	0.97 (0.73, 1.28)	1.01 (0.76, 1.34)	1.28 (0.93, 1.76)	0.17
2-naphthalene	1 (referent)	1.02 (0.80, 1.31)	1.18 (0.86, 1.62)	**1.33 (1.03, 1.72)**	1.40 (0.99, 1.96)	0.008
2-fluorene	1 (referent)	1.09 (0.83, 1.43)	1.03 (0.76, 1.40)	1.25 (0.91, 1.73)	1.22 (0.90, 1.73)	0.15
3-fluorene	3-fluorene*BMI interaction					
1-phenanthrene	1 (referent)	0.88 (0.69, 1.13)	1.00 (0.75, 1.33)	1.10 (0.80, 1.52)	1.23 (0.88, 1.73)	0.09
2-phenanthrene	1 (referent)	0.86 (0.65, 1.14)	1.14 (0.82, 1.60)	1.17 (0.88, 1.56)	1.33 (0.99, 1.78)	0.02
3-phenanthrene	1 (referent)	0.89 (0.67, 1.18)	0.86 (0.63, 1.19)	1.02 (0.73, 1.43)	1.11 (0.82, 1.51)	0.30
1-pyrene	1 (referent)	**0.72 (0.54, 0.96)**	0.78 (0.58, 1.05)	1.03 (0.77, 1.37)	1.06 (0.77, 1.47)	0.21

Weighted and adjusted for age, sex, poverty index ratio, ethnicity, BMI, smoking status and urinary creatinine

Abbreviations: OR, odds ratio; 95% CI, 95% confidence interval

**Fig 1:**
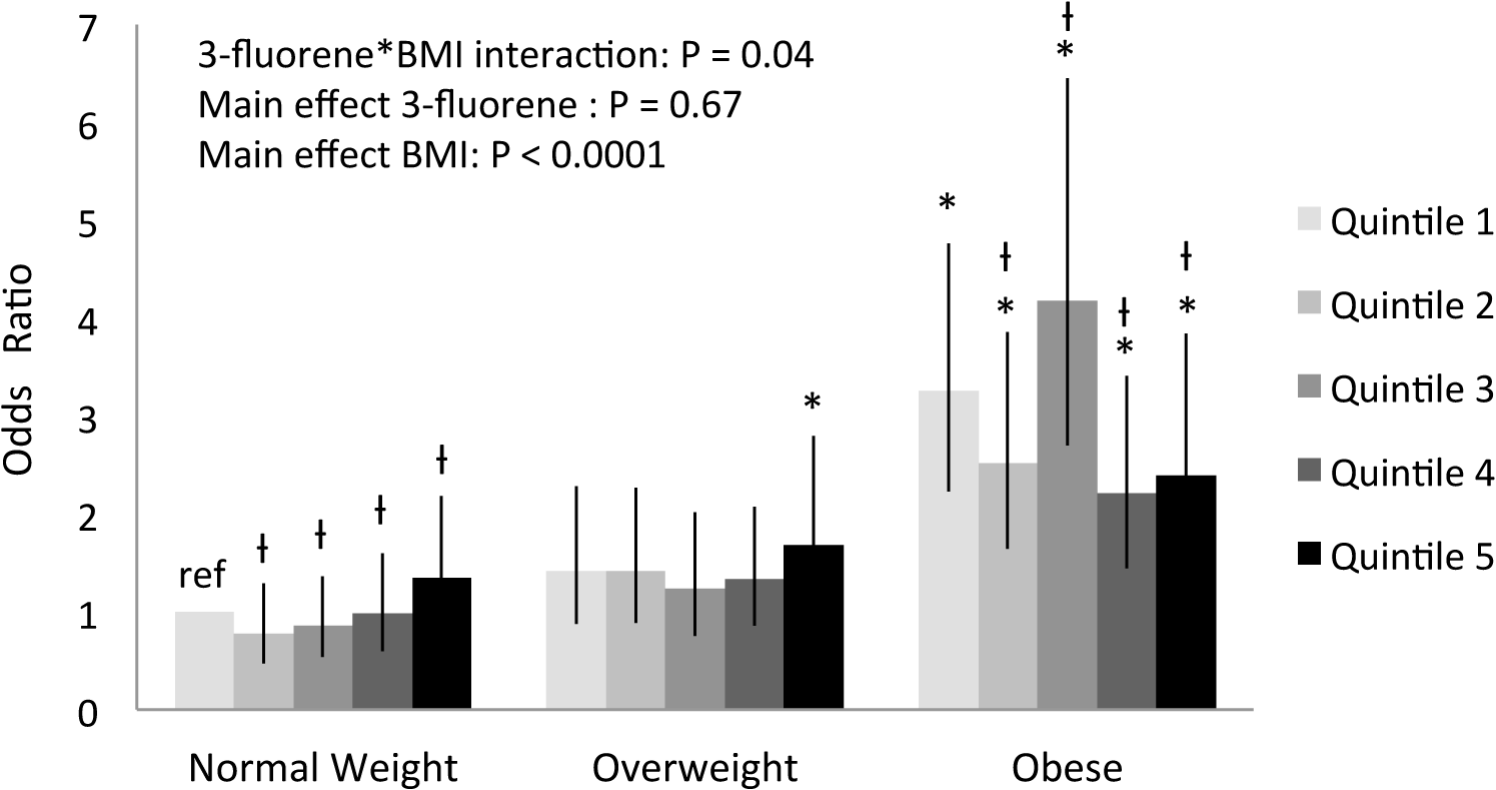
Weighted and adjusted odds ratio for hypertension and 3-fluorene quintiles by weight status. Weighted and adjusted for age, sex, poverty income ratio, ethnicity, smoking status and urinary creatinine. * Significantly different from normal weight in the lowest 3-fluorene quintile (reference group), P≤0.05. Ɨ Significantly different from the lowest 3-fluorene quintile within BMI category P≤0.05

A positive dose-response relationship was observed with 2-naphthalene (P_trend_ = 0.008) and 2-phenanthrene (P_trend_ = 0.02) for hypertension. An interaction effect was observed for hypertension (3-fluorene x BMI interaction, P = 0.04). In specific high levels of 3-fluorene had a deleterious influence on hypertension (**Fig 1**). Individuals with obesity had a greater odds ratio for prevalent hypertension than normal weight individuals in the lowest quintile of 3-fluorene. Within the individuals with obesity, those in quintile 3 had the highest odds of hypertension (P < 0.0001).

No interaction effects were observed for T2D (PAH x BMI interactions, P > 0.05). For a given BMI, individuals in the highest quintiles of 1 and 2-naphthalene had an 82% (P = 0.05) and 124% (P = 0.02) greater odds ratio of prevalent T2D respectively (**Table 4**) compared to the lowest quintiles. In addition, for a given BMI, those in the highest quintiles of 2-phenanthrene and 1-pyrene were 78% (P = 0.006) and 80% (P = 0.001) more likely to have of T2D than those in the lowest quintile respectively. A significant negative dose-dependent association with T2D was observed for quintiles of 1-naphthalene (P_trend_ = 0.05) and 2-naphthalene (P_trend_ = 0.02), while 1- and 2-phenanthrene and 1-pyrene were positively associated with T2D (P_trend_ = 0.01, P_trend_ = 0.006, and P_trend_ = 0.001 respectively).

**Table 4:** Weighted and adjusted odds ratios for quintiles of PAH, and type 2 diabetes and dyslipidemia.

	Type 2 Diabetes					
	Quintile 1	Quintile 2	Quintile 3	Quintile 4	Quintile 5	*P*_trend_
*Metabolite*:		OR (*95% CI*)	OR (*95% CI*)	OR (*95% CI*)	OR (*95% CI*)	
1-naphthalene	1 (referent)	1.17 (0.77, 1.78)	1.16 (0.78, 1.72)	1.08 (0.72, 1.62)	**1.82 (1.15, 2.89)**	0.05
2-naphthalene	1 (referent)	**1.81 (1.07, 3.05)**	**1.67 (1.04, 2.70)**	1.38 (0.88, 2.16)	**2.24 (1.45, 3.48)**	0.02
2-fluorene	1 (referent)	1.15 (0.78, 1.68)	1.17 (0.68, 1.99)	1.07 (0.69, 1.67)	1.52 (0.97, 2.39)	0.15
3-fluorene	1 (referent)	0.93 (0.65, 1.33)	1.14 (0.72, 1.80)	0.82 (0.55, 1.23)	1.36 (0.87, 2.13)	0.39
1-phenanthrene	1 (referent)	0.83 (0.56, 1.24)	1.14 (0.80, 1.63)	1.51 (0.91, 2.51)	1.41 (0.94, 2.11)	0.01
2-phenanthrene	1 (referent)	1.61 (0.77, 1.74)	1.32 (0.85, 2.04)	1.44 (0.93, 2.24)	**1.78 (1.17, 2.71)**	0.006
3-phenanthrene	1 (referent)	1.07 (0.76, 1.49)	1.27 (0.88, 1.83)	1.09 (0.71, 1.68)	1.23 (0.81, 1.86)	0.37
1-pyrene	1 (referent)	1.14 (0.77, 1.69)	1.33 (0.91, 1.95)	1.47 (0.94, 2.29)	**1.80 (1.22, 2.64)**	0.001
	**Dyslipidemia**					
1-naphthalene	1 (referent)	1.17 (0.92, 1.48)	0.96 (0.74, 1.24)	1.19 (0.94, 1.50)	**1.38 (1.05, 1.80)**	0.03
2-naphthalene	1 (referent)	1.03 (0.79, 1.34)	1.09 (0.81, 1.45)	1.20 (0.99, 1.45)	**1.54 (1.20, 1.97)**	0.0003
2-fluorene	1 (referent)	1.00 (0.80, 1.24)	1.01 (0.80, 1.29)	1.13 (0.90, 1.41)	**1.57 (1.21, 2.05)**	0.001
3-fluorene	1 (referent)	1.11 (0.85, 1.45)	0.99 (0.74, 1.32)	1.00 (0.78, 1.30)	**1.68 (1.28, 2.21)**	0.01
1-phenanthrene	1 (referent)	0.86 (0.66, 1.13)	1.10 (0.88, 1.38)	0.95 (0.72, 1.25)	1.16 (0.86, 1.56)	0.24
2-phenanthrene	1 (referent)	1.07 (0.87, 1.33)	1.19 (0.96, 1.48)	**1.29 (1.02, 1.64)**	**1.47 (1.08, 2.01)**	0.009
3-phenanthrene	1 (referent)	0.96 (0.76, 1.20)	1.23 (0.98, 1.55)	1.11 (0.86, 1.42)	1.27 (0.96, 1.67)	0.05
1-pyrene	1 (referent)	0.90 (0.73, 1.12)	1.05 (0.82, 1.34)	1.26 (0.94, 1.67)	1.30 (0.96, 1.77)	0.01

Weighted and adjusted for age, sex, poverty index ratio, ethnicity, BM, smoking status and urinary creatinine; Abbreviations: OR, odds ratio; 95% CI, 95% confidence interval

For dyslipidemia (**Table 4**), no interaction effects were observed (PAH x BMI interactions, P > 0.05). All the PAH metabolites were positively associated in a dose-dependent manner with the odds for prevalent dyslipidemia (P_trend_ ≤ 0.05), except for 1-phenanthrene (P_trend_> 0.05). After adjustments for confounders, those in the highest quintiles of 1- and 2-naphthalene had 38% (P = 0.03) and 54% (P = 0.0003) greater odds of prevalent dyslipidemia than the lowest quintile respectively. In addition, those in the highest quintiles of 2- and 3-fluorene and 2-phenanthrene were 57% (P = 0.001), 68% (P < 0.01) and 47% (P = 0.009) more likely to have dyslipidemia compared to the lowest quintiles. There were no significant associations observed for dyslipidemia and 1-phenanthrene (P > 0.05).

## Discussion

To our knowledge, we are one of the first to examine the association between PAH and obesity in adults. In addition, we are one of the first to examine the relationship between PAH and 3RFMetS, diabetes, hypertension, and dyslipidemia independent of BMI. We observed various urinary PAH biomarkers to have significant deleterious associations with a number of different health risk independent of BMI, however these dose-dependent associations were observed to be both positive and negative depending on the PAH metabolite and health factor. These findings bring to light the potentially detrimental impact of certain urinary PAH biomarkers on obesity and metabolic conditions in the general population.

Literature on the relationship between obesity and PAH in the general population has been limited to date [16,25]. A study on prenatal exposure to PAH report that children whose mothers had high PAH exposure during pregnancy were at significantly greater risk for obesity compared to children whose mothers were exposed to lower levels [25]. Similarly, another study on children report that naphthalene metabolites are significantly associated with BMI [16]. Contrary to previous studies on children [25] we observed PAH metabolites to be both negatively and positively associated with obesity depending on the PAH metabolite. Though no causal relationship can be extrapolated here, the association between obesity and PAH may suggest that PAH is a modest contributor to the increasing prevalence of obesity or may highlight the metabolic differences between children and adults.

In line with previous research, we observe that naphthalene [26] and phenanthrene metabolites are associated with a greater risk of T2D [26,27]. We extend these observations by demonstrating this relationship between 1-pyrene and T2D independent of BMI. Therefore, PAH may exacerbate the already high risk for T2D associated with obesity and may be a cause for concern. Considering the health consequences commonly related with T2D [28], these findings suggest further research on the health effects of PAH are warranted.

To our knowledge, no studies have examined the relationship between hypertension and PAH in the general population. We observed that, for a given BMI, those with the highest levels of 2-naphthalene and 2-phenanthrene biomarkers were more likely to have hypertension, while the association between 3-fluorene and prevalent hypertension in individuals with obesity may be curvilinear. These novel results raise questions about the potential mechanisms of how PAH modify hypertension risks. A study on rodents suggests that PAH may result in carcinogenesis and atherogenesis by increasing inflammation and the size of plaques in arteries [29]. However, more research examining the mechanisms of PAH on hypertension in the general population and in specific, the obese population is needed.

Although studies on potential PAH and hypertension relationships are nearly non-existent, one recent study investigated the effects of environmental pollutants on lipid markers. In general, this study reports high levels of fluorene biomarkers to be associated with unfavorable HDL levels [30]. In the current study, we observed that compared to low levels, those with high levels of naphthalene, fluorene and 2-phenanthrene were 38–68% more likely to have dyslipidemia, for a given BMI. These findings are consistent with the previous study as both observed PAH to be related to a disadvantageous lipid profile [30], however, we also observed the naphthalene biomarkers and 2-phenanthrene to be related to dyslipidemia whereas no such findings were demonstrated in previous literature. It is important to note that the present study observed PAH to be associated with the condition of dyslipidemia as a whole, which uses cut-offs that have been proven to increase the risk of cardiac-related illnesses. Therefore, this method of measurement may be a better indicator of risk as opposed to isolating each component. These differences may also be why we observe certain PAH metabolites to be associated with a greater risk of 3RFMetS for a given BMI. MetS is recognized to be related with numerous risk factors including CVD [31], which is becoming of increasing relevance since more than 20% of the U.S. adult population are affected by MetS [32]. This highlights the notion that common environmental exposures such as PAH may influence the expression of obesity-related health risk, and warrants further investigation.

We are aware of a number of potential limitations of the present study. Although there are currently no standard reference material to compare the accuracy of urinary PAH measurements, the labelling of internal standards with isotopes is known to provide precise measurements [18]. This study employs a cross-sectional design and therefore does not allow for the extrapolation of causal or temporal relationships between PAH and health risk. In addition, examined urinary concentrations of PAH are more reflective of immediate or short-term PAH exposure [1] and do not account for the PAH levels stored in fatty tissue. Therefore we still require studies to investigate PAH levels in fat tissue as it relates to health risk over an extended period of time. The urinary metabolites being assessed in this study are reliable indicators of recent exposure to PAH [23,33,34]. However the examination of these eight metabolites is due to the availability of data in our opportunistic sample, and may not be the PAH metabolites that are most closely related with CVD and diabetes risk. Further, whether or not the metabolites of previously established toxic and carcinogenic PAHs including benzo[a,e]pyrene and chrysene [1] have similar effects on cardiometabolic health remains unknown. Nevertheless, an important strength of this study is our ability to demonstrate the association between metabolic conditions and PAH, independent of BMI using a large sample size. In addition, all multivariable statistics models were adjusted for smoking status, which allowed us to account for the underlying variability caused by cigarette smoke inhalation. Furthermore, these results are representative and generalizable to the general U.S. adult population.

In conclusion, PAH is associated with greater risk of cardiometabolic health conditions independent of BMI. Adults with high levels of PAH may have greater or lower risk of obesity, 3RFMetS, hypertension, dyslipidemia and T2D depending on the PAH metabolite. This suggests future studies should explore the physiological mechanisms of each biomarker on health.
